# High Expression of Cathepsin E in Tissues but Not Blood of Patients with Barrett’s Esophagus and Adenocarcinoma 

**DOI:** 10.1245/s10434-014-4155-y

**Published:** 2014-10-28

**Authors:** Oliver M. Fisher, Angelique J. Levert-Mignon, Sarah J. Lord, Natalia K. Botelho, Araluen K. Freeman, Melissa L. Thomas, Dan Falkenback, Antony Wettstein, David C. Whiteman, Yuri V. Bobryshev, Reginald V. Lord

**Affiliations:** 1 St. Vincent’s Centre for Applied Medical Research, Sydney, Australia; 2 NHMRC Clinical Trials Centre, University of Sydney, Sydney, Australia; 3 Department of Epidemiology and Medical Statistics, School of Medicine, University of Notre Dame, Sydney, Australia; 4 Department of Surgery, Lund University Hospital (Skane University Hospital) and Lund University, Lund, Sweden; 5 Diagnostic Endoscopy Centre, St. Vincent’s Clinic, Sydney, Australia; 6 QIMR Berghofer Medical Research Institute, Brisbane, Australia; 7 Faculty of Medicine, School of Medical Sciences, University of New South Wales, Sydney, Australia; 8 Department of Surgery, School of Medicine, University of Notre Dame, Sydney, Australia

## Abstract

**Background:**

Cathepsin E (CTSE), an aspartic proteinase, is differentially expressed in the metaplasia–dysplasia–neoplasia sequence of gastric and colon cancer. We evaluated CTSE in Barrett’s esophagus (BE) and cancer because increased CTSE levels are linked to improved survival in several cancers, and other cathepsins are up-regulated in BE and esophageal adenocarcinoma (EAC).

**Methods:**

A total of 273 pretreatment tissues from 199 patients were analyzed [31 normal squamous esophagus (NE), 29 BE intestinal metaplasia, 31 BE with dysplasia (BE/D), 108 EAC]. CTSE relative mRNA expression was measured by real-time polymerase chain reaction, and protein expression was measured by immunohistochemistry. CTSE serum levels were determined by enzyme-linked immunosorbent assay.

**Results:**

Median CTSE mRNA expression levels were ≥1,000-fold higher in BE/intestinal metaplasia and BE/D compared to NE. CTSE levels were significantly lower in EAC compared to BE/intestinal metaplasia and BE/D, but significantly higher than NE levels. A similar expression pattern was present in immunohistochemistry, with absent staining in NE, intense staining in intestinal metaplasia and dysplasia, and less intense EAC staining. CTSE serum analysis did not discriminate patient groups. In a uni- and multivariable Cox proportional hazards model, CTSE expression was not significantly associated with survival in patients with EAC, although CTSE expression above the 25th percentile was associated with a 41 % relative risk reduction for death (hazard ratio 0.59, 95 % confidence interval 0.27–1.26, *p* = 0.17).

**Conclusions:**

CTSE mRNA expression is up-regulated more than any known gene in Barrett intestinal metaplasia and dysplasia tissues. Protein expression is similarly highly intense in intestinal metaplasia and dysplasia tissues.

**Electronic supplementary material:**

The online version of this article (doi:10.1245/s10434-014-4155-y) contains supplementary material, which is available to authorized users.

Barrett’s esophagus (BE) is the condition in which the normal distal squamous lining of the esophagus is replaced by specialized metaplastic columnar epithelium.^1^ BE is the strongest recognized risk factor for esophageal adenocarcinoma (EAC), a highly malignant cancer with an unparalleled 6-fold increase in incidence over the past three decades.^2^ Less than 5 % of patients presenting with EAC have a previous diagnosis of BE because they have not undergone endoscopy, but even for patients under surveillance, there are significant problems, including sampling error and difficulties with the histopathologic interpretation of degree of dysplasia, and EAC may develop between endoscopies.^3^^–^ 5

These problems have stimulated the search for clinically relevant biological markers, but so far, no biomarkers have proven sufficient for routine clinical practice.^6^,^7^ Because of the problems with endoscopic diagnosis and surveillance, it is worth exploring non endoscopic, cheaper, and less-invasive diagnostic and monitoring options, such as blood, saliva, and brush cytology tests.

Cathepsin E (CTSE) is an intracellular aspartic protease that is normally expressed in a wide range of immune cells but also is present in osteoclasts and gastric epithelial cells; secreted forms have been described.^8^^–^ 10 A differential expression pattern has been demonstrated for CTSE in normal, metaplastic, dysplastic, and neoplastic gastric epithelium as well as in the intestinal dysplasia–neoplasia sequence in APC^min/+^ mice.^11^^–^ 15 Furthermore, CTSE has also been suggested as both a diagnostic and a prognostic biomarker for some cancers.^16^

Other members of the cathepsin family (cathepsin B, C, D, K, and S) have been found to be up-regulated in BE and EAC, but analysis of CTSE in BE and EAC has not been reported.^17^^–^ 20

This study aimed to evaluate the potential prognostic value of CTSE to predict progression to more advanced disease in patients with Barrett metaplasia–dysplasia–adenocarcinoma spectrum, and to predict survival for patients with EAC.

## Materials and Methods

### Study Design, Study Population, and Specimen Collection

A diagnostic case control analysis was performed to examine the associations among the following: (1) CTSE tissue mRNA expression and normal squamous esophagus (NE), BE, BE with dysplasia (BE/D) and EAC; (2) CTSE serum protein levels and NE, BE, BE/D and EAC; and (3) the association between CTSE and overall EAC patient survival (Supplementary Fig. 1).

**Fig. 1:**
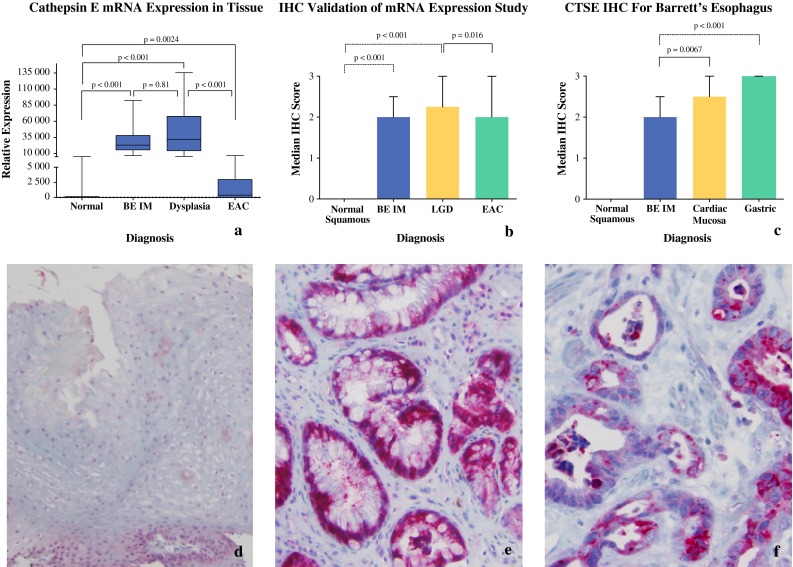
CTSE mRNA expression analysis (**a**). CTSE is increased in BE and BE/D in comparison to NE. CTSE mRNA expression of CTSE in EAC is significantly lower than BE and dysplastic BE, but is significantly higher compared to NE. Immunohistochemical staining for CTSE (**d**–**f**). Marked, intense staining in BE and EAC with decreased staining intensity and shift in staining pattern with neoplastic progression of Barrett epithelium. Quantification of these findings could be statistically confirmed (**b**, **c**)

The NE, BE, and BE/D tissues as well as blood samples were collected at endoscopies performed at St. Vincent’s Hospital, Sydney, from patients prospectively enrolled onto a research collaboration entitled PROBE-NET (Progression of Barrett’s Esophagus to Esophageal Adenocarcinoma Network). The EAC specimens were obtained either from patients at St. Vincent’s Hospital or from patients who had been enrolled onto the population-based case-control Australian Cancer Study.^21^ All tissues were fixed in formalin and embedded in paraffin. The pathology diagnosis was established by pathologists at the respective host institutions. Before mRNA extraction, a section of each tissue sample was also sent for hematoxylin and eosin staining and reviewed to confirm the pathology in the research specimen. BE was defined as intestinal metaplasia with the presence of goblet cells. Patient serum samples were collected at study recruitment, centrifuged at 14,000× *g,* and then stored at −80 °C until further use.

For the analysis of CTSE as a prognostic marker for EAC survival, we used tissue samples from an independent cohort of 75 patients with early stage EAC (I–IIB) from the Australian Cancer Study.^21^ All subjects had undergone treatment with potentially curative surgery alone and received no chemo- or radiotherapy. Patients who died within 30 days of surgery or who had cancer-involved operative resection margins (R1/R2 resection) were excluded.

Institutional review board approval for this study was obtained at all collaborating institutions, and all patients provided written informed consent.

### RNA Isolation

From each paraffin-embedded tissue block, two 7 μm sections were cut and used for RNA extraction using the Qiagen FFPE RNeasy Kit (Cat. #74404; Qiagen, Valencia, CA) following the manufacturer’s protocol. RNA yield and quality was measured using a Biospec Nano spectrophotometer (Shimadzu Scientific Instruments, Sydney, Australia).

### Multiplexed Tandem Polymerase Chain Reaction

Multiplexed tandem polymerase chain reaction (MT-PCR) was used to quantitate the mRNA expression level of CTSE and a reference gene, *NONO* (non-POU domain containing, octamer-binding (NONO), transcript variant 2; NM_007363), using the Rotor-Gene 6000 real-time quantitative PCR system (Corbett Life Sciences/Qiagen, Sydney, Australia), as described previously.^22^,^23^ Primers were designed with the help of Primer 3 software modified by AusDiagnostics Pty. Ltd. (AusDiagnostics, Alexandria, New South Wales, Australia), leading to a CTSE “inner” amplicon of 73 bp and an “outer” amplicon of 120 bp. Outer primer sequences for CTSE were 5′-CTCAATGGACCAGAGTGCCAAG-3′ (forward) and 5′-GAGGAGCCAGTGTCGAAGATG-3′ (reverse). Inner primer sequences were 5′-GAGTGCCAAGGAACCCCTCATC-3′ (forward) and 5′-TGGTGGGGAGCCAATGGAGATA-3′ (reverse). All primer pairs spanned an intron–exon boundary, and all samples were run in duplicate. The correct size and integrity of the products was verified on a Bioanalyzer DNA separation chip (Agilent Technologies, Forest Hill, Victoria, Australia).

### CTSE Enzyme-Linked Immunosorbent Assay

A CTSE enzyme-linked immunosorbent assay (ELISA) kit (Biomatik Corp, Cambridge, Ontario, Canada) was used to measure CTSE protein levels in serum. Briefly, after reconstitution of all reagents, serum samples were incubated on precoated plates at 37 °C and 70 % humidity for 2 h. After addition of the primary antibody and incubation for another hour at 37 °C, plates were washed three times with wash buffer. Addition of the secondary antibody was followed by a further incubation for 30 min at 37 °C, and plates were then washed another five times before the addition of the reaction substrate. For antibody binding detection, the supplier’s detection reagent was added for 15 min and the reaction halted by addition of the provided stopping solution. Plate readouts occurred in a 96-well multiplate reader (Multiskan Microplate Reader; Thermo Labsystems/Thermo Scientific, Waltham, MA) at an absorbance of 450 nm. All samples were assayed in triplicate and run without dilution. All plate readings had an intra-assay coefficient of variation <15 %.

### Immunohistochemistry

Tissue specimens were processed in a standard fashion with regular formalin fixation and paraffin embedding. CTSE was identified in 5 µm tissue sections using a rabbit polyclonal anti-CTSE antibody (Cat. #ab36996; Abcam, Waterloo, NSW, Australia) in a standard alkaline phosphatase anti-alkaline phosphatase technique, as described previously.^24^

### Immunohistochemistry Scoring

The sections were scored using a four-step scale: (0) no staining or equal to background, (1) weak diffuse cytoplasmic staining, (2) moderate cytoplasmic staining in at least 10 % of cells, and (3) strong immunostaining in a majority of cells.^25^ Immunohistochemistry sections were scored by two experienced investigators who were blinded to clinical information. In cases of disagreement, consensus was reached after reanalysis on a multiheaded microscope.

### Statistical Analysis

The mRNA raw expression values were obtained on the Rotor-Gene MT-PCR system, and then relative expression values were calculated as the ratio of the mRNA level of CTSE to the control gene *NONO,* with the expression of *NONO* set to a fixed level (1000). Where necessary, log_2_ transformation of relative expression values and/or serum values was performed to achieve normal distribution. Differences between two groups were measured by Student’s *t* test or the Wilcoxon rank-sum test. One-way analysis of variance was used to compare differential gene and protein expression between patient groups. The Kaplan–Meier method was used for survival estimates, and differences in survival were analyzed using the log-rank test. Cox proportional hazards models were used for uni- and multivariable analysis. All *p* values of ≤0.05 were regarded as statistically significant. All analyses were performed by the SAS Statistical Package, version 9.2 (SAS Institute, Cary, NC). Prism (GraphPad Prism version 6.0c for Mac OS X; GraphPad Software, San Diego, CA) was used for graphs.

## Results

### Patients and Tissues

As shown in Table 1, a total of 273 tissue specimens from 199 patients were included. Ninety-one patients were studied to evaluate CTSE as a marker for the progression of BE to EAC, 33 patients provided serum samples to evaluate CTSE as a biomarker in blood, and 75 early-stage EAC patients were included in the evaluation of CTSE as a prognostic biomarker. Each part of the study included an independent cohort of patients, thus allowing for intrastudy validation of CTSE as a marker for the respective pathologies. Despite chart review, the correct tumor stage could not be assessed in 8 patients (7.4 %) as a result of incomplete clinical data.

**Table 1:** Clinical and pathologic characteristics of included patients

Characteristic	All (*n* = 199)		BE progression study (*n* = 91)		Serum analysis (*n* = 33)		Prognostic biomarker in EAC (*n* = 75)	
	*n*	%	*n*	%	*n*	%	*n*	%
Sex								
Male	164	82.4	67	73.6	29	87.9	68	90.7
Female	35	18.6	24	26.4	4	12.1	7	9.3
Age, year, median (IQR)	63	(55–71)	62	(53–69)	63	(54–70)	67	(59–74)
Diagnosis								
Healthy controls/normal squamous	31	15.6	22	24.2	9	27.3	–	–
BE intestinal metaplasia	29	14.6	21	23.1	8	24.2	–	–
BE with dysplasia	31	15.6	22	24.2	9	27.3	–	–
Esophageal adenocarcinoma	108	54.2	26	28.5	7	21.2	75	100
TNM (AJCC, 7th edition)								
Tis	2	1.9	2	7.7	–	–	–	–
T1–2	88	81.5	8	30.7	5	71.4	75	100
T3–4	8	7.4	6	23.1	2	28.6	–	–
N1–3	34	31.5	7	26.9	3	42.9	24	32.0
M+	3	2.8	3	11.5	–	–	–	–
Unknown T, N or M	10^a^	9.3	10^a^	38.5	–	–	–	–
Tumor stage (AJCC, 7th edition)								
0 (Tis)	2	1.9	2	7.7	–	–	–	–
IA–B	33	30.6	2	7.7	3	42.9	28	37.3
IIA	26	24.1	2	7.7	1	14.3	23	30.7
IIB	29	26.9	4	15.4	1	14.3	24	32.0
IIIA–C	7	6.5	5	19.2	2	28.6	–	–
IV	3	2.8	3	11.5	–	–	–	–
Unknown stage	8^a^	7.4	8^a^	30.8	–	–	–	–
Survival, d, median (range)	1,182	(630–1,685)	1,305	(195–1,780)	1,277	(766–1,342)	1,161	(724–1,663)

Totals may not equal 100 % due to rounding

*BE* Barrett’s esophagus, *EAC* esophageal adenocarcinoma, *IQR* interquartile range, *TNM* tumor, node, metastasis classification system, *AJCC* American Joint Committee on Cancer

^a^Two patients included had no clinical data on T and N status but were found to be M+ at assessment. Regardless of this, primary tissue samples were used for analysis in the respective study. These patients were excluded from survival analysis

#### Expression Analysis

As shown in Fig. 1a, median CTSE mRNA relative expression was more than 1,000-fold higher in BE compared to NE (18.41 vs. 23,221; *p* < 0.001). Median CTSE mRNA expression in EAC was lower than in BE and dysplastic BE (*p* < 0.001) but higher than in NE (875.14 vs. 18.41; *p* = 0.0024).

#### Immunohistochemistry

All BE specimens and all EAC specimens stained strongly for CTSE, with high specificity to the glandular structures and almost absent staining of the stromal fraction of the esophageal specimens. Figure 1d–f provides representative immunostaining patterns of the respective histopathology tissue types.

CTSE staining was completely absent within the squamous epithelium, whereas in BE/D median staining scores were 2.25 (*p* < 0.001, Fig. 1b). Staining intensities in EAC were similar to BE (median staining score 2.0), but the location shifted more apically and the staining pattern showed more granular features in EAC. Staining in EAC was significantly lower than in BE/D (2.0 vs. 2.25; *p* = 0.016, Fig. 1b).

CTSE immunostaining was also assessed in cardiac and gastric fundus mucosa because CTSE is known to be present in gastric glands.^13^ CTSE staining was significantly higher in both proximal gastric mucosae compared to BE (2.0 vs. 2.5 and 3.0; *p* = 0.0067 and <0.001, respectively; Fig. 1c).

#### CTSE Serum ELISA

**Fig. 2:**
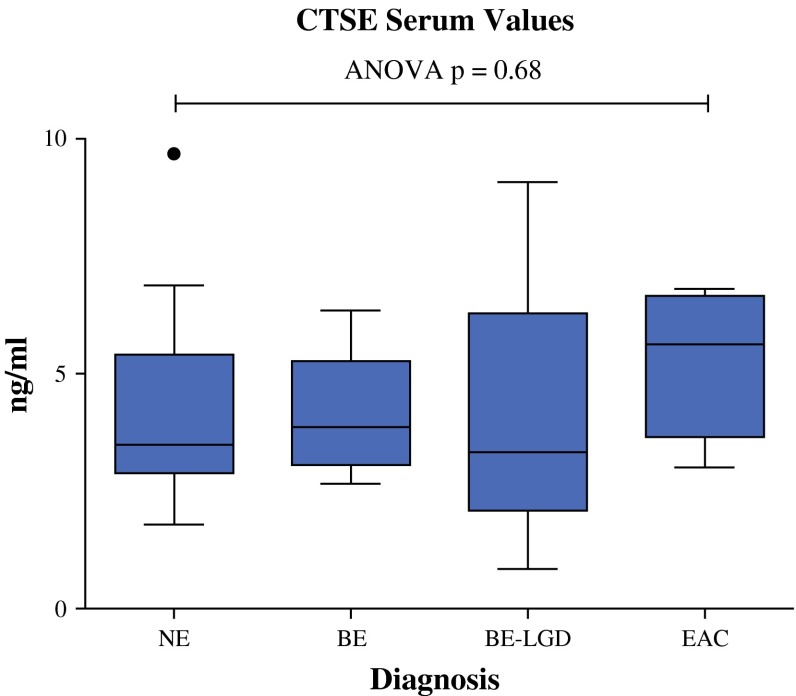
CTSE serum values. Serum values do not differ between pathologic patient groups, although tissue levels are markedly increased

As shown in Fig. 2, there were no significant differences between patient groups.

### Analysis of CTSE as a Prognostic Biomarker in Early-Stage EAC

#### Patient Survival

Overall median survival of the patients in the independent EAC cohort was 3.2 years (38.7 months), and overall 5-year survival was 66 %.

T1a and T1b patients showed a significantly increased survival (58.7 and 46.0 months, respectively) compared to T2 patients (25.2 months; *p* < 0.001). Stage I (IA+B) patients had a median survival of 41.2 months (3.4 years), whereas stage II (IIA+IIB) survived 31.4 months (2.61 years, *p* = 0.0027).

### CTSE mRNA Expression and Tumor Stage

No significant difference was found in CTSE expression levels between T stages, American Joint Committee on Cancer (AJCC) stage I versus II cancers, lymph node negative versus positive disease, or male versus female sex (data not shown).

#### CTSE EAC Tissue mRNA Expression and Survival

**Fig. 3:**
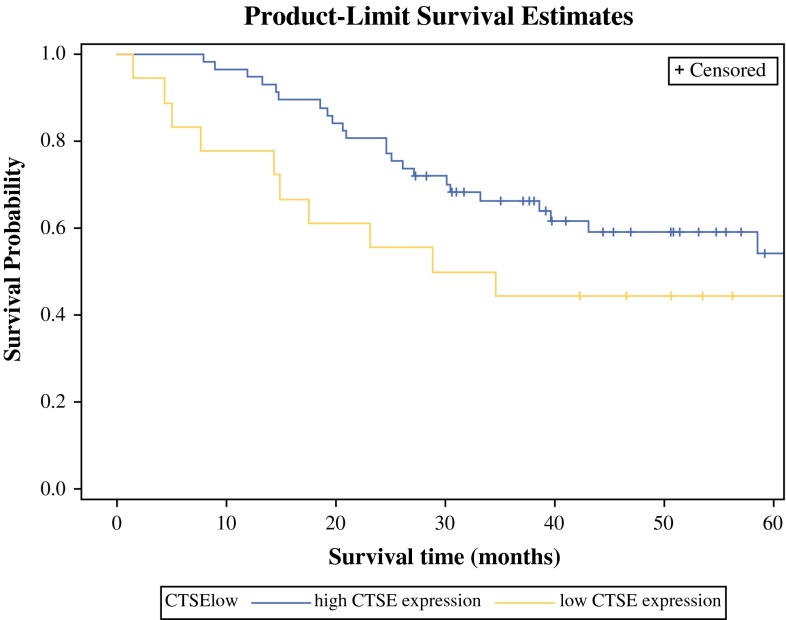
CTSE mRNA expression and survival. Dichotomization at log_2_-transformed 25th percentile. Patients with CTSE over the 25th percentile show a 41 % reduction in risk for death (HR 0.59, 95 % CI 0.27–1.26; *p* = 0.17)

For survival analysis, CTSE expression values were dichotomized at the 25th percentile, median, and 75th percentile to determine the influence of CTSE expression on overall patient survival. Kaplan–Meier survival curves showed that patients with a CTSE expression above the 25th percentile had a non-significant trend toward improved overall survival (log-rank *p* = 0.14, Fig. 3). In uni- and multivariable analysis, elevated CTSE expression levels were not significantly associated with survival [hazard ratio (HR) 0.65; 95 % confidence interval (CI) 0.73–3.24, *p* = 0.25). CTSE expression above the 25th percentile was associated with a non-significant 41 % relative risk reduction for death (HR 0.59, 95 % CI 0.27–1.26, *p* = 0.17). In a backward stepwise regression model including sex, age, overall tumor stage, and CTSE expression below the 25th percentile, only age (HR 1.04, 95 % CI 1.00–1.08; *p* = 0.04) and AJCC stage II (HR 4.93, 95 % CI 1.88–12.88; *p* = 0.001) were independent prognostic markers for decreased survival (Table 2).

**Table 2:** Uni- and multivariable analysis for factors contributing to mortality according to the Cox proportional hazards model

Variable	Univariable analysis			Multivariable analysis		
	HR	95 % CI	*p*	HR	95 % CI	*p*
Age	1.04	1.00–1.08	0.04	1.04	1.00–1.08	0.04
Sex (male)	1.71	0.41–7.15	0.464	1.49	0.35–6.38	0.59
AJCC stage II	4.68	1.80–12.14	0.002	4.93	1.88–12.88	0.001
Log CTSE <25th percentile	1.54	0.73–3.24	0.25	1.71	0.79–3.65	0.17
Log CTSE >25th percentile	0.65	0.31–1.36	0.25	0.59	0.27–1.26	0.17

*HR* hazard ratio, *CI* 95 % confidence interval, *AJCC* American Joint Committee on Cancer, *CTSE* cathepsin E

## Discussion

This novel study shows that CTSE is highly overexpressed in BE and BE/D compared to normal esophageal tissue. CTSE mRNA expression was 1,000-fold higher in BE compared to normal esophageal tissue, which we believe to be the highest gene expression change reported for this disease. Lower levels of CTSE mRNA were observed in EAC compared to BE. A similar expression pattern has been demonstrated for CTSE in normal, metaplastic, dysplastic, and neoplastic gastric epithelium as well as in the intestinal dysplasia–neoplasia sequence in APC^min/+^ mice.^11^^–^ 15 One explanation for our observed CTSE overexpression is that exposure to gastric refluxate induces expression of gastric proteases; consistent with this we found that despite the remarkable induction in BE tissues, CTSE levels are still lower than those found in either gastric fundus or cardiac mucosa.

Alternatively, the remarkably high CTSE expression levels may indicate a functional role in this disease. Unfortunately, the exact function of CTSE remains to be defined because the specific substrate for this protease is not known.^10^,^26^ A role in host defense has been suggested because of the high CTSE expression in immune and antigen presenting cells, but CTSE was solely expressed in glandular BE cells and not in stromal cells in the present study, similar to the distribution observed in a mouse intestinal neoplasia study.^14^

Interestingly, the other cathepsin family members cathepsin B, C, K, and S are also up-regulated in BE and EAC, and cathepsin D (CTSD) mRNA expression shows a significant stepwise increase in erosive esophagitis, intestinal metaplasia and EAC.^17^^–^ 20 Further functional hypotheses for CTSE in Barrett disease involve the contents of the gastro-esophageal refluxate. Intracellular and secreted CTSD requires a low pH to exert its proteolytic activity, leading to the speculation that CTSD activity may be especially enhanced in the acidic environment of gastroesophageal reflux associated disease.^20^,^27^ CTSD is also involved in the resistance to the bile salt deoxycholate–induced apoptosis in colon cancer cell lines.^28^ Because CTSE is highly homologous to CTSD, there may be a similar acid and bile-associated function for CTSE in the context of BE development.^9^,^26^

A conclusive explanation for the significantly lower CTSE expression levels in EAC compared to BE is not available. In particular, it is not clear if CTSE expression is down-regulated in EAC and thus CTSE more a marker of BE than of EAC. In other cancers, CTSE has been shown to exert an antitumorigenic effect in prostate cancer cells—for example, by acting as the cleavage enzyme for tumor necrosis factor-related apoptosis ligand (TRAIL), which has also been implicated in the pathogenesis of EAC.^29^,^30^ Injection of purified CTSE into human tumor xenografts results in a dose-dependent induction of apoptosis and inhibition of tumor growth.^29^ It can therefore be speculated that the increased levels of CTSE in BE and BE/D may serve a protective mechanism. In this hypothesis, the down-regulation of CTSE marks an enhanced susceptibility to neoplasia formation, as suggested by a mouse melanoma study.^29^ Further, loss of CTSE expression has also been shown to induce mammary gland neoplasia.^31^

High levels of CTSE have been shown to be associated with improved survival in various cancers, but in EAC we found only a non-significant trend in which expression of CTSE above the 25th percentile resulted in a 41 % risk reduction for death.^31^^–^ 33A larger study including patients with worse disease stage could be undertaken, as limited power and small survival differences due to the inclusion of only early stage, chemoradiotherapy-naive patients may have reduced our ability to detect a statistically significant association.

Finally, although highly desirable from a clinical perspective, this study indicates a lack of value in measuring CTSE protein levels in serum. Although there was a non-significant trend to higher CTSE protein levels in patients with EAC, our exploratory study was not powered to detect small differences in CTSE expression between patient groups. Alternatively, however, CTSE activity levels could be studied according to a recent report, which showed that CTSE activity levels but not protein levels were associated with more advanced disease, recurrence, and prognosis in patients with breast cancer.^31^

## Conclusions

The remarkable induction of CTSE expression in BE intestinal metaplasia and dysplasia, together with the significant down-regulation in EAC tissues, suggests a possible role for CTSE in the Barrett disease spectrum. The intense CTSE protein expression in BE and lower levels of expression in EAC could be evaluated by pathologists as a method to simplify the evaluation of esophageal tissues, although we acknowledge that further studies are required to substantiate this potential benefit.

## Electronic supplementary material

Below is the link to the electronic supplementary material.

Summary flow chart of cathepsin E analysis performed in this study (DOCX 59 kb)
