# Supplementary material for: High Expression of Cathepsin E in Tissues but Not Blood of Patients with Barrett’s Esophagus and Adenocarcinoma
Source: Ann Surg Oncol. 2014 Oct 28;22(7):2431–8. doi: 10.1245/s10434-014-4155-y (PMC4458267; doi:10.1245/s10434-014-4155-y)
Supplement: Supplementary file 1 — Summary flow chart of cathepsin E analysis performed in this study (DOCX 59 kb) [file 10434_2014_4155_MOESM1_ESM.docx]

**Supplementary Figure 1. Study Synopsis**
